# Advances in scanning probe microscopy for biological systems

**DOI:** 10.52601/bpr.2025.250023

**Published:** 2026-08-31

**Authors:** Songping He, Xiaodong Yu, Weixing Li, Wei Ji, Qing Huan

**Affiliations:** 1 Beijing National Laboratory for Condensed Matter Physics, Institute of Physics, Chinese Academy of Sciences, Beijing 100190, China; 2 University of Chinese Academy of Sciences, Beijing 100049, China; 3 Institute of Biophysics, Chinese Academy of Sciences, Beijing 100101, China; 4 Key Laboratory for Vacuum Physics, University of Chinese Academy of Sciences, Beijing 100190, China

**Keywords:** Scanning probe microscope, Nucleic acids, Proteins, Cells

## Abstract

Scanning probe microscopy (SPM), as a nanoscale characterization technique, employs a sharp probe to detect local tip-sample interactions through near-field physical phenomena. This approach achieves atomic-resolution surface imaging while enabling concurrent characterization of multi-parametric properties — electrical, magnetic, and chemical signals. This review offers a cross-disciplinary perspective on the advances in SPM for biological systems, which serves as a practical guide for life scientists to select from the expanding array of SPM techniques. We outline the fundamental principles of scanning tunneling microscopy (STM) and atomic force microscopy (AFM), before discussing a series of advanced SPM techniques: force spectroscopy for nanomechanical characterization, Kelvin probe force microscopy (KPFM) for surface potential imaging, scanning near-field optical microscopy (SNOM) for super-resolution optics, tip-enhanced Raman spectroscopy (TERS) for nanoscale chemical identification, and scanning electrochemical microscopy (SECM) for localized electrochemical activity detection. A systematic comparison of these technologies provides researchers with clear criteria to select the optimal methodology for diverse demands, either characterizing nucleic acids and proteins or analyzing single-cell ultrastructure and biomechanics. In addition, this review explores the transformative integration of SPM and artificial intelligence (AI). This integration is expected to automate SPM workflows. It will also increase the stability of SPM systems and enhance the reproducibility of experimental results. Furthermore, by addressing current challenges and future perspectives of *in vivo* imaging, this review aims not merely to review the progress but to empower biologists to harness these intelligent multi-modal SPM systems for groundbreaking discoveries.

## INTRODUCTION

The earliest optical microscopes first enabled humans to observe bacteria and cells, laying the foundation for microscopic analysis. This tradition of discovery advanced decisively when, in the same year, the Nobel Prize recognized two revolutionary instruments: the electron microscope (EM) and the scanning tunneling microscope (STM), the latter a member of the scanning probe microscopy (SPM) family. Together, these breakthroughs have fundamentally reshaped how we probe the structure and properties of matter and living systems. The electron microscope primarily includes two types: scanning electron microscopy (SEM) and transmission electron microscopy (TEM). The core principle of SEM involves scanning a focused beam of electrons across the sample surface. Detectors collect secondary electrons or backscattered electrons generated by electron-sample interactions, which are used to reconstruct three-dimensional surface images and compositional information. TEM, on the other hand, transmits a high-energy electron beam through an ultrathin sample. The transmitted electrons form an image that reveals the internal structure of the specimen. In contrast to both, SPM does not use an electron beam for imaging. Instead, it relies on a physically sharp tip at the atomic level that scans point by point across the surface, measuring interaction forces or quantum tunneling currents between the tip and the sample to reconstruct surface topography.

It's hard to study wet biological samples with normal electron microscopes because the vacuum sucks out the water, and the electron beam damages samples, ruining their natural structure. Cryo-electron microscopy (cryo-EM) overcomes these limitations by rapidly vitrifying samples into amorphous ice, preserving near-native architecture under vacuum and mitigating radiation damage. This capability enables high-resolution three-dimensional reconstructions and was recognized by the 2017 Nobel Prize in Chemistry. Today, cryo-EM is central to structural biology and drug discovery: it has resolved pathogens such as Zika virus and SARS-CoV-2, providing structural foundations for vaccines and antivirals (Sirohi *et al*. 2016; Yan *et al*. 2020), and it routinely resolves challenging targets — including ion channels, membrane receptors, and dynamic molecular machines such as the ribosome (Liu *et al*. 2018; Ma *et al*. 2022; Zhang *et al*. 2017). By delivering near-atomic views in functional contexts, cryo-EM continues to transform our understanding of life.

Although cryo-EM holds a central position for resolving near-atomic structures in biology, its need for high vacuum and the beam sensitivity of specimens means it is better at providing “frozen” structural snapshots. It still has limits for *in-situ* studies under conditions close to physiology and for fast, dynamic processes. Complementary to cryo-EM, SPM provides a well-balanced solution to these characterization requirements. Unlike techniques that rely on high-energy electron beams, which require high vacuum and cause inevitable sample damage through ionization and radical formation, SPM utilizes a physical probe that scans the sample surface to detect local interactions — such as van der Waals forces, mechanical contact, or tunneling currents. The invention of these techniques brought major breakthroughs to materials and surface science. STM exploits quantum tunneling for atomic-scale imaging and enables single-atom manipulation and studies of confined electronic states (Binnig *et al*. 1983; Crommie *et al*. 1993; Eigler and Schweizer 1990). AFM overcomes STM’s constraint to conductive samples, achieving atomic resolution on insulators, and operating in liquids (Binnig *et al*. 1986; Hansma *et* *al*. 1993). Conventional materials are rigid, periodic, and stable in dry or vacuum conditions. SPM has been widely used to characterize topological materials, manipulate atoms, and perform local spectroscopy. Whereas biological matter is soft, highly hydrated, and dynamic, with interactions that shift with pH and ionic strength. These contrasts demand liquid-phase, low-perturbation measurements that resolve nanoscale structure, local mechanics, and weak molecular interactions. Accordingly, functionalized SPM now fits this need, the accessible sample space has expanded from inorganic, hard solids to organic and soft matter, enabling multi-parameter measurements on biological specimens — structure, mechanics, interactions, and chemical signals — so that SPM now extends naturally into in situ studies in the life sciences. As a crucial complement to cryo-EM, the cornerstone technique for nanoscale characterization in biology, SPM is continuously expanding the dimensions of bio-characterization.

In this review, we analyze the fundamental working principles of various SPM technologies, including their operational modes and key performance parameters. The SPM family has continued to expand and evolve through technological convergence, encompassing a wide range of techniques including STM, AFM, force spectroscopy, Kelvin probe force microscopy (KPFM), scanning near-field optical microscopy (SNOM), tip-enhanced Raman spectroscopy (TERS), and scanning electrochemical microscopy (SECM), continually inspiring new biological research applications. We discuss their strengths and limitations, with a focus on how these characteristics influence their suitability for specific biological applications. Current challenges and limitations of SPM technologies are also addressed, with the aim of providing biological researchers with practical references for technology selection and application.

## PRINCIPLES OF SPM TECHNIQUES

This section elaborates on the fundamental principles of various SPM techniques and their representative application scenarios within biological systems. Building upon this foundation, a systematic comparative analysis will be conducted to delineate the distinctive advantages and inherent limitations of each SPM modality in addressing diverse biological inquiries.

### Scanning tunneling microscopy and atomic force microscope

As core techniques of SPM, STM and AFM serve as the foundation for many advanced SPM derivatives.

**Figure 1:**
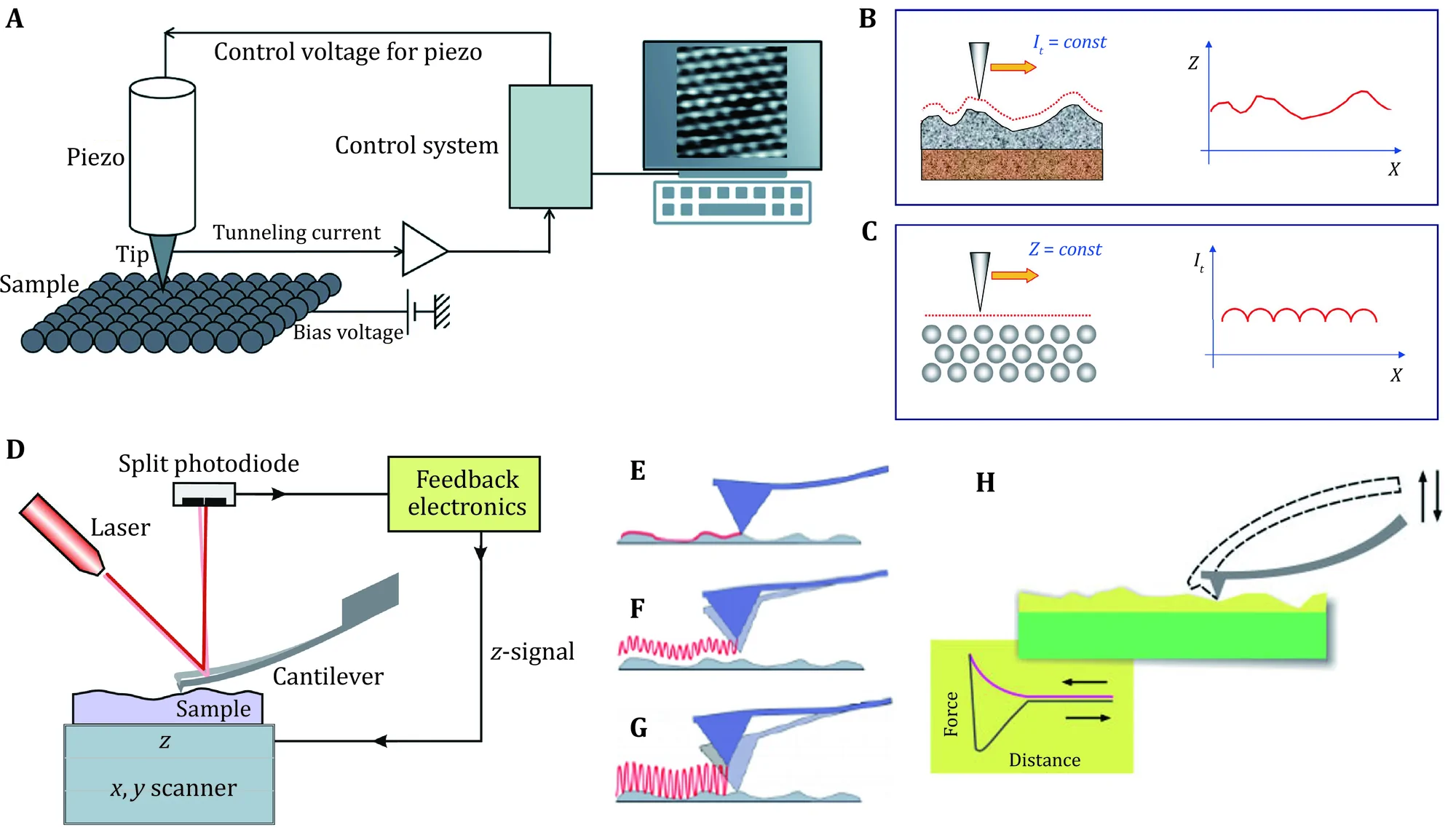
Schematic diagram of the working principles of STM and AFM. **A** Schematic diagram of the STM system (Yang and Freund 2024). **B**,**C** STM constant current mode imaging (**B**) and constant height mode imaging (**C**) (Mironov 2014). **D** Schematic diagram of AFM (Voigtländer 2015). **E**−**G** Contact mode of AFM (**E**), tapping mode of AFM (**F**), and non-contact mode of AFM (**G**) (McKay 2021). **H** Force spectroscopy mode of AFM (Liu and Wang 2010)

STM works based on quantum tunneling. When the distance between a sample and the probe tip is reduced to the nanometer scale, applying a bias voltage between them generates a tunneling current. The tunneling current is formed by electrons jumping between the tip and the sample. This current is extremely sensitive to the tip-sample separation, changing exponentially with the distance. By measuring this current, we can directly generate topographic images of the sample surface (Fig. 1A). STM typically operates in two modes: constant current mode (Fig. 1B), in which a feedback loop adjusts the vertical position of the tip to maintain a constant tunneling current during the scan. The recorded adjustments in the tip's height directly map the surface topography. Constant height mode (Fig. 1C), in which the tip is scanned at a fixed height above the sample, and the resulting variations in the tunneling current are recorded. These current variations correspond to the surface topography.

The invention of STM brought a major breakthrough to materials science, enabling direct observation of surface reconstructions, defects, and adsorbates, as well as the characterization of nanomaterials such as graphene, carbon nanotubes, and quantum dots. Scanning tunneling spectroscopy (STS) is the key functional extension of STM: it augments the purely spatial coordinates (*x*, *y*, *z*) with an energy axis (E), thereby visualizing the spatial distribution of electronic states at selected energies — that is, the spatial form of the electronic wave function (Pavarini *et* *al*. 2016; Wang *et al*. 2006). In superconductivity research, STS has become indispensable. Upon entering the superconducting state, Cooper pairing opens a superconducting gap near the Fermi level and modifies the single-particle excitation spectrum; STS can directly resolve this gap, providing clear and definitive experimental evidence of superconducting behavior (Ruby *et al.* 2015).

However, the application of STM in biology is significantly limited. Most biological macromolecules are non-conductive, which prevents the generation of a tunneling current and restricts STM analysis primarily to conductive specimens (Driscoll *et al*. 1990; Rodríguez-Galván and Contreras-Torres 2022). Furthermore, ultra-high vacuum (UHV) is often necessary for stable imaging, which prevents biological samples from maintaining their natural function. And achieving reliable images under physiological liquids remains a challenge.

AFM overcomes the primary limitation of STM, because it does not require sample conductivity. Instead of measuring tunneling current, AFM senses interatomic forces, enabling it to image samples in various environments, including air and liquid. The most widely used AFM employs the laser reflection method, with its representative schematic structure depicted in (Fig. 1D). The core component is the force sensor — typically a microfabricated cantilever capable of oscillation. The cantilever has a sharp probe tip at one end. As the probe interacts with the surface, interatomic forces cause the cantilever to deflect or alter its vibrational state. A laser beam reflected from the cantilever onto a photodetector measures these minute changes. This signal is then processed to generate a map of physical properties that the sample has, most commonly its topography. This capability allows researchers to directly visualize and quantify a wide range of nanoscale surface features — for example, atomic steps on crystalline surfaces and single nanoparticles (Darwich *et al*. 2011; Pramanick *et al*. 2009). Beyond imaging, AFM can apply and measure minute forces to quantitatively probe materials’ nanoscale mechanical responses, enabling the extraction of parameters such as Young’s modulus, adhesion, and friction (Enrriques *et al*. 2022). Unlike traditional AFM, which uses a flexible cantilever and an optical system to detect minute changes in deflection, the qPlus-AFM employs a specially designed rigid quartz tuning fork as its core sensing element. The "q" stands for Quality Factor, which reflects the degree of energy dissipation of a system during resonance. A much higher q (qPlus) value means that the vibration of the tuning fork attenuates slowly and has higher force sensitivity, thereby achieving high precision, compared to conventional AFM cantilevers. This technology always works in a UHV and low-temperature environment (Giessibl 2003).

Cantilever-based AFM operates in three primary modes: contact mode, tapping mode, and force spectroscopy.

In contact mode (Fig. 1E), the probe tip continuously touches the sample as it scans. At distances typically within 2–3 Å of a sample surface, short-range repulsive forces dominate the interaction between an AFM probe and the material. These forces arise from quantum mechanical effects — primarily Pauli exclusion and Coulombic repulsion between electrons. A feedback system will automatically adjust the scanner's height. This keeps the cantilever's deflection constant, such that the applied force stays the same. The height data collected during this process are then used to create a topographical map of the sample.

In non-contact mode (Fig. 1F), the cantilever also oscillates at or near its resonant frequency, but the probe tip does not touch the sample surface. The probe maintains a small distance from the surface, typically 1–10 nm. This mode primarily senses weak attractive forces, such as van der Waals forces. Although van der Waals forces are the weakest among long-range forces, they exhibit strong dependence on probe–sample distance (*r*), scaling with *r*^−7^. This high sensitivity makes them the primary force measured in non-contact AFM mode for generating surface images. As the probe approaches the surface, these forces change the frequency and amplitude of the cantilever's oscillation. A feedback loop is used to maintain a constant oscillation parameter (either frequency or amplitude). The changes in the *Z*-axis position are then recorded to construct an image of the sample's topography.

Tapping mode (Fig. 1G), also known as intermittent-contact mode, is better suited for delicate samples. In tapping mode, the cantilever doesn't scrape across the surface. Instead, it oscillates rapidly, usually at or near its natural resonance frequency. This rapid oscillation means the tip just barely "taps" the surface of the sample, making quick, brief contacts. By monitoring changes in this oscillation, we can gather information about the surface's topography. During the majority of the oscillation cycle, long-range van der Waals attractive forces dominate. At the instant of closest approach to the sample, short-range Pauli repulsive forces become predominant. The method is fantastic because it substantially reduces lateral forces, which is so important for preventing damage to really delicate samples like nucleic acids, proteins, and cells.

### Force spectroscopy

The emergence of force spectroscopy, based on AFM, has brought a revolutionary breakthrough to the detection of physical forces. It is the only disruptive tool capable of precisely quantifying, imaging, and manipulating the mechanical properties of samples with piconewton (pN) force resolution and nanometer spatial resolution. In force spectroscopy (Fig. 1H), the probe is moved vertically toward and away from a single point on the sample surface, and the cantilever deflection is recorded to generate a force−distance (F−D) curve. This curve provides detailed information about tip-sample interactions. Force spectroscopy not only provides mechanical parameters and adhesion/friction forces of materials and interfaces (Butt *et al*. 2005; Cappella and Dietler 1999), but also resolves energy barriers and dynamics at the single-molecule level (Dudko *et al.* 2006, 2008; Evans and Ritchie 1997), and spatially maps these quantities through force spectroscopy imaging (Radmacher *et* *al*. 1994).

Force spectroscopy is an extremely powerful tool in virology research. Its core advantage lies in its ability to perform nanoscale imaging and mechanical manipulation of individual viral particles under near-physiological conditions, thereby compensating for the shortcomings of traditional techniques. In virology research, force spectroscopy experiments are primarily divided into two fundamental modes: nanoindentation and single-molecule/single-virus force spectroscopy. The former involves vertically pressing an AFM probe onto a single viral particle fixed on a substrate, measuring the mechanical response of the virus by recording the relationship between force and indentation depth. This method can directly obtain information such as the stiffness, elasticity, and mechanical failure points of the viral capsid (Hernando-Pérez *et al*. 2019; Najera *et al*. 2023; Ray *et al*. 2025). The latter involves chemically functionalizing a single viral particle or viral protein onto an AFM probe, then allowing the functionalized probe to contact and retract from a surface with corresponding receptors (such as living cells or functionalized substrates). By analyzing rupture events recorded in the retraction curve, the specific dissociation forces between molecules can be quantified (Ray *et* *al*. 2025).

Force-volume imaging (FVI) is an advanced atomic force microscopy mode designed to overcome the limitations of single-point force spectroscopy. It extends force spectroscopy measurements from a single point to an area, generating a two-dimensional distribution map of the mechanical or adhesive properties of the sample surface. In this mode, a complete F–D curve is systematically acquired at each point across a predefined two-dimensional grid, generating a three-dimensional dataset. This dataset contains mechanical properties — such as adhesion, stiffness, and elasticity — at each (*X*, *Y*) coordinate, which can be directly correlated with the corresponding topographic information (Olubowale *et al*. 2021). The key value of FVI lies in its ability to combine spatial and mechanical data to produce spatial maps of mechanical properties across a sample. This capability is especially important for studying the heterogeneity of biological specimens. Using FVI, researchers can visually identify significant variations in mechanical parameters — such as stiffness or adhesion — across different regions of the same cell or tissue. As a result, FVI has become a powerful tool for analyzing complex systems like biomaterials, cells, and tissues at the nanoscale, offering new perspectives for understanding their structure–function relationships (Kontomaris *et al*. 2023).

Beyond virology, force spectroscopy is now a routine way to quantify nanoscale interactions and mechanics in biology — covering receptor–ligand binding (Evans and Ritchie 1997; Williams 2003), protein/nucleic-acid unfolding (Janovjak *et al*. 2003), and cell adhesion and elasticity (Viljoen *et al*. 2021).

### Kelvin probe force microscopy

**Figure 2:**
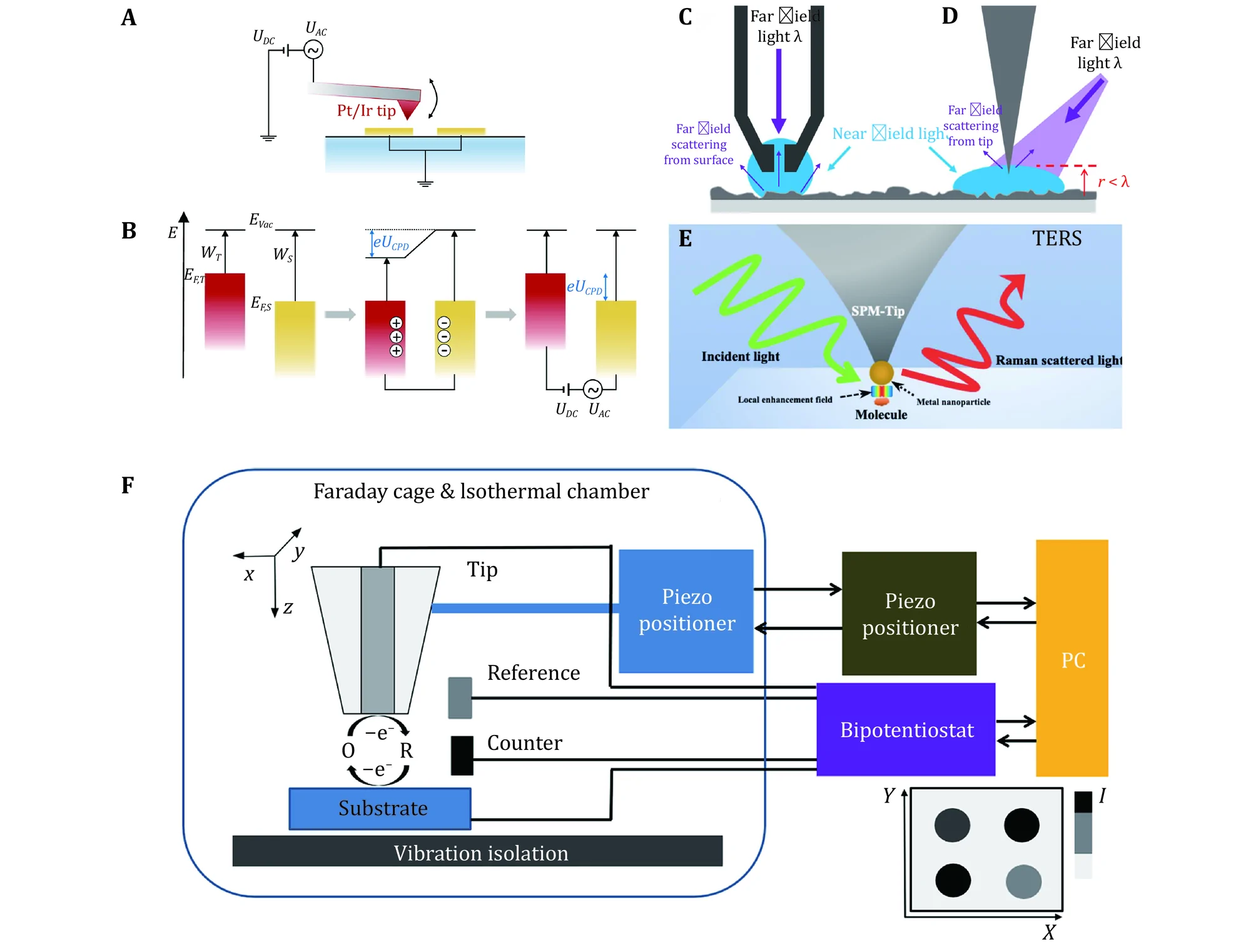
Schematic diagram of the different principles of SPMs. **A**,**B** Concept of Kelvin probe force microscopy. A Pt/Ir coated KPFM probe tip is scanned over the structure (gold) while a voltage with DC component *U*_*DC*_ and AC component *U*_*AC*_ is applied to the tip (**A**). Tip (red) and structure (gold) have different work functions *W*_*T*_ and *W*_*S*_ (**B**). Contact-driven equilibration forms *U*_*CPD*_, bringing Fermi levels into alignment. *U*_*CPD*_ is obtained by adding *U*_D*C*_ and *U*_A*C*_ until the force associated with the electric field is offset. *E*_*F*_,_*T*_: tip fermi energy; *E*_*F*_,_*S*_: sample fermi energy (Ochs 2022). **C**,**D** The near-field and types of SNOM tips. Aperture SNOM, where an evanescent light field created by a nanoscale opening at the end of a tip is scattered off the surface (**C**). Light scattered from the surface is detected in the far-field. Apertureless SNOM utilizes near-field light emitted from the surface due to external illumination that is scattered off of a sharp tip into the far-field to be detected (**D**) (Bazylewski *et al*. 2017). **E** Schematic tunnelling-controlled TERS in a confocal-type side-illumination configuration, in which *V*_b_ is the sample bias and *I*_t_ is the tunnelling current (Zhang *et* *al*. 2013). **F** Schematic diagram of SECM. The electrochemical measurement system consists of a four-electrode electrochemical cell with a working electrode for the probe, a working electrode for the sample substrate, a reference electrode, a counter electrode, and a bipotentiostat to control the potential and current of the two working electrodes (Bae 2024)

KPFM is an advanced AFM technique that maps the surface potential or work function of samples with high spatial resolution. It operates by measuring the contact potential difference (CPD). When the probe is brought within nanometers of the sample, electrons move between them until their Fermi levels align, and establish a CPD between the two materials. To measure the CPD, a bias voltage is applied to the probe to nullify this electrostatic force. The voltage required to cancel the force is equal to the CPD (Figs. 2A and 2B), providing a direct map of the local surface potential. KPFM enables quantitative nanoscale surface-potential mapping that reveals work function, doping, and band bending in semiconductors and 2D materials (Robinson *et al*. 2018; Yoon *et* *al*. 2020; Rothhardt *et* *al*. 2024), resolves phase separation, grain-boundary energetics, and surface photovoltage in photovoltaic/optoelectronic systems (Fuchs *et al*. 2016; Nie *et* *al*. 2024; Qin *et* *al*. 2021), and probes electrostatic screening and temperature-dependent behavior in ferroelectric and other polar materials (Segura *et al*. 2013; Shvebelman *et al*. 2002).

The core application of KPFM in the biological field lies in its ability to detect the surface potential distribution of biological systems and processes in a label-free and non-destructive manner. These electrical potentials are closely related to the charge state of biomolecules, their functional activity, and interfacial phenomena. A potential difference exists across the cell membrane (membrane potential), which is fundamental to cellular signal transduction, energy production (mitochondria), and excitability (neurons, muscle cells). KPFM can non-invasively measure the surface potential of individual living cells from the outside, reflecting their physiological state. Biomolecules such as proteins, DNA, and lipids often carry a net charge under physiological conditions. Their structure and function are highly dependent on charge distribution and electrostatic interactions (Tsai *et al*. 2012). By monitoring changes in the electrical potential distribution, it is possible to gain insights into the mechanisms of biomolecular interaction (Birkenhauer and Neethirajan 2014; Rahimi *et al*. 2025). In the fields of biosensors, organic electronic devices (*e*.*g*., implantable devices), and biomimetic materials, the interfacial potential at the material-biology interface is critical in determining their performance and biocompatibility (Gest *et al*. 2024). With the assistance of KPFM, the applicability and stability of biomaterials and devices can be significantly enhanced.

### Scanning near-field optical microscopy

The resolution of conventional optical microscopy is limited by Abbe's diffraction limit, which dictates that features smaller than roughly half the wavelength of light cannot be distinguished. This is because traditional microscopes collect only propagating light waves that travel into the far field. However, light-matter interactions also generate evanescent waves — non-propagating electromagnetic fields that are confined to a "near-field" region close to the sample. These waves contain high-resolution, sub-wavelength information but decay exponentially with distance, making them undetectable with conventional optics.

SNOM, developed by Pohl *et al*, brilliantly overcomes this barrier (Pohl *et al*. 1984). By positioning a nanoscale tip within this evanescent wave-dominated near-field zone, SNOM converts localized optical near fields into measurable signals, enabling nanoscale spatial resolution. Practically, it integrates a laser source, scanning probe, detector, and piezoelectric positioning, and — especially for biological samples — benefits from close coupling with AFM. In aperture SNOM (a-SNOM), a metal-coated tapered fiber with a ~60–100 nm subwavelength aperture confines and delivers light to the sample (Fig. 2C). In apertureless SNOM (s-SNOM), a focused laser illuminates a (often metallized) AFM tip that functions as a nano-antenna, producing a strongly confined, locally enhanced near field at the tip apex (Fig. 2D); the resulting elastic scattering from the tip–sample near-field interaction is collected in the far field. SNOM enables label-free, sub-diffraction imaging and spectroscopy, supporting real-space mapping of polaritons and guided modes (Li *et al*. 2015b), nano-IR chemical identification (Chen *et al*. 2019), visualization of nanoscale carrier and vibrational dynamics in semiconductors and two-dimensional materials (Nishida *et al*. 2022), interrogation of biological and soft-matter systems (Umakoshi 2023), and quantitative reconstruction of near-field distributions in nanophotonic devices (Bazylewski *et al*. 2017; Stanciu *et al*. 2022).

By pushing optical resolution into the nanometer domain, SNOM provides a unique capacity to visualize and characterize individual biomacromolecules and their assemblies. This capability addresses a key limitation by integrating atomic-resolution structural data from methods like X-ray crystallography with the ensemble cellular context provided by traditional cell biology. It allows researchers to study the structure and organization of life's building blocks in a context that more closely resembles their functional environment. SNOM has been successfully employed to image individual DNA molecules, providing direct visualization of their topography on a substrate (Kirsch *et al*. 1997). And this technology has been used to confirm the successful creation of protein patterns using a combination of nanoimprint lithography and molecular assembly (Falconnet *et al*. 2004). A key revolutionary application of SNOM in cell biology is the label-free chemical mapping of intracellular components using s-SNOM and its nano-Fourier Transform Infrared spectroscopy (nano-FTIR) variant. This approach probes the intrinsic vibrational signatures of molecules, eliminating the need for fluorescent labels or stains, which can perturb the very biological processes under investigation (Greaves *et al*. 2023; Hu *et* *al*. 2023).

The chemical sensitivity of s-SNOM has significant potential in clinical applications, particularly in cytopathology and cancer diagnosis. The progression of cancer is often accompanied by changes in cellular biochemistry, such as an increased synthesis of proteins, lipids, and nucleic acids to support rapid proliferation. A combination of s-SNOM with a high-power, tunable infrared free electron laser (IR-FEL) has been shown to be a promising tool for detecting cervical cancer. The technique was able to distinguish between normal cervical cells and those with low-grade or high-grade dyskaryosis by identifying specific changes in their infrared spectra (Halliwell *et al*. 2016). Beyond animal cells, SNOM is also proving to be a valuable tool in plant biology (Keplinger and Burgert 2020). The complex structure of plant cell walls, composed primarily of lignocellulosic materials, is critical for both the plant's structural integrity and for industrial applications like biorefining. As a non-invasive technique, SNOM's key advantage lies in its ability to simultaneously acquire both topographic and optical images at a spatial resolution that surpasses the conventional diffraction limit.

### Tip-enhanced Raman spectroscopy

TERS is a powerful technique that merges SPM with the chemical fingerprinting capability of surface-enhanced Raman spectroscopy (SERS). Pioneered as a concept by Wessel, TERS provides both topographical and chemical information simultaneously and with extremely high resolution. The core principle is based on generating localized surface plasmons. Figure 2E illustrates the diagram of TERS. A laser is focused onto a sharp, metal-coated AFM tip, creating a highly concentrated electromagnetic field at its apex. This "hotspot" dramatically amplifies the Raman signals from molecules directly beneath the probe. By scanning the tip point-by-point across a sample, TERS produces hyperspectral maps that reveal chemical composition with a spatial resolution of just a few nanometers — far beyond the diffraction limit. TERS provides label-free chemical fingerprinting with nanoscale — often single-molecule — sensitivity (Kang *et al*. 2022), enabling quantitative mapping of defects, strain, and doping in 2D and semiconductor materials (Huang *et al*. 2019). Because of its exceptional sensitivity and chemical specificity, TERS enables correlative structural and chemical analysis at the nanoscale, making it a highly valuable tool for characterizing complex biological specimens. TERS enables in-depth analysis of the chemical composition and structure of nucleic acids. It can identify characteristic Raman spectra of nucleotides (adenine-A, guanine-G, cytosine-C, thymine-T) and additionally probe vibrational modes originating from the nucleic acid backbone, such as deoxyribose and phosphate groups (Bonhommeau *et al*. 2022).

One of the most compelling applications of TERS lies in its potential for label-free DNA sequencing. Studies have demonstrated that using a “gap-mode” configuration with a silver tip and a gold substrate enables TERS to achieve sub-nanometer spatial resolution below 1 nm. In this approach, a single-stranded DNA (ssDNA) molecule is stretched and immobilized on a gold surface, and a silver-coated tip is scanned along it, acquiring TERS signals at steps as small as 0.5 nm. By analyzing the characteristic Raman peaks of specific nucleotides within the measured spectra, the DNA base sequence can be identified nucleotide by nucleotide (He *et al*. 2019; Rasmussen and Deckert 2006).

This method represents a fundamental breakthrough in label-free, direct nucleic acid sequencing, offering a stark contrast to conventional techniques that require complex labeling and amplification steps. Its applications span from label-free DNA sequencing and analysis of protein secondary structures to nanoscale imaging of chemical heterogeneity in viral strains and cellular membranes (Mrđenović *et al*. 2022; vandenAkker *et al*. 2016).

### Scanning probe electrochemical microscopy

Developed by Allen J. Bard and colleagues in the 1980s, SECM is a powerful technique that creates spatially resolved maps of chemical reactivity at the surface of samples. It operates within an electrolyte solution, using an ultramicroelectrode (UME) as its probe. These probes are small-scale electrodes, typically fabricated from electroactive materials such as platinum, gold, or carbon, sealed within an insulating sheath of glass or another polymer. The active region is polished flat to create a disk-shaped electrode with a well-defined diameter, often in the micrometer to nanometer range. A diagram of the experimental setup is provided in Fig. 2F. As the UME scans in close proximity to the sample, it measures Faradaic currents from localized redox reactions, providing a direct image of electrochemical activity (Bard *et al*. 1989). SECM provides quantitative, high-resolution images of electrochemical processes at interfaces. It is uniquely capable of mapping local chemical kinetics and catalytic activity, as well as visualizing concentration gradients and flux. This makes it an essential tool for diagnosing issues like corrosion and coating defects, for testing the performance of batteries and electrocatalysts under operating conditions, and for monitoring chemical signals at the interfaces of individual cells (Bard *et al*. 1989; Shi *et* *al*. 2020).

Imaging cell surface morphology and activity represents one of the most classic applications of SECM. This technique involves scanning a UME over the surface of cells and measuring changes in the redox reaction rates of electroactive species (such as oxygen, ascorbic acid, neurotransmitters, *etc*.) near the cell surface. These measurements reflect cellular metabolic activity or membrane permeability (Bard *et al*. 2006; Conzuelo *et* *al*. 2018; Filice and Ding 2019).

In addition to surface imaging, SECM can be employed to detect the enzymatic activity of immobilized enzymes or on cell surfaces, and to facilitate the development of highly sensitive biosensors. By immobilizing enzymes on a substrate, specific reactions catalyzed by these enzymes can generate or consume electroactive species. The SECM probe quantitatively measures the activity of the enzyme by detecting changes in the concentration of these electroactive substances (Shiku *et al*. 1996; Shen *et* *al*. 2022). By positioning the SECM probe near neurons or clusters of nerve cells, it becomes possible to monitor the dynamics of neurotransmitter release (such as dopamine and adrenaline) in real time and with high spatiotemporal resolution under stimulating conditions. This represents a highly valuable application of SECM in neuroscience (Kurulugama *et al*. 2005; Shen *et* *al*. 2018).

Modern SECM is often combined with AFM. In this combined system, the AFM maps the surface shape and controls the probe's position. At the same time, the micro-electrode measures the chemical activity at the exact same spot. This integrated approach allows for the direct correlative mapping of a surface's physical structure and its chemical reactivity. Consequently, SECM is an invaluable tool for applications such as corrosion science, catalyst evaluation, and biological sensing (*e*.*g*., monitoring ions and neurotransmitters at the cellular level) (El-Giar and Wipf 2007).

### Comparison of SPM techniques

The scientific community needs to probe the physical, chemical, and mechanical properties of many different samples. This demand has led to a steady expansion of SPM techniques. Experts and scholars from various fields have collaboratively developed these methods. Some SPM techniques haven’t been used in biology yet. However, these techniques offer unique measurement characteristics, showing great potential for biological applications. Scanning microscopy techniques such as ballistic electron emission microscopy (BEEM) (Kaiser and Bell 1988) based on STM can be used to probes buried electronic interfaces, spin-polarized scanning tunneling microscopy (SP-STM) (Wiesendanger *et al*. 1990, 1991) designed for mapping electron spin polarization, scanning hall probe microscope (SHPM) (Oral *et al*. 1996) used to quantify magnetic field distribution, synchrotron X-ray scanning tunneling microscopy (SX-STM) (Chiu *et al*. 2008; Rose *et al*. 2013) used to correlate synchrotron X-ray spectroscopy with atomic topography, multi-probe STM (Nakayama *et al*. 2012) used to perform concurrent multi-point electrical measurements. Table 1 systematically compares the core similarities and differences among SPM techniques for biological applications, providing researchers with critical selection criteria to identify the optimal methodology aligned with their specific research requirements.

**Table 1:** Comparison of different SPM technologies

Technology	Advantages	Limitations	Sample requirements	Lateral/ longitudinal resolution	Imaging speed	Reference
STM	Atomic-resolution electronic property probing, suitable for conductive biomolecules	Not applicable to insulating samples, high sample preparation requirements	The sample/substrate must be electrically conductive	< 0.1 nm/0.01 nm	Minute-scale per image acquisition	Rodríguez-Galván and Contreras-Torres 2022
AFM	Imaging and mechanical measurements of living cells, proteins, DNA, *etc*. under near-physiological conditions	May cause damage to soft specimens during contact mode operation	No conductivity requirement	1−5 nm/0.1−0.5 nm	Minute-Scale per image acquisition high-speed AFM < 100 ms/frame	Xia and Youcef-Toumi 2022
KPFM	Label-free mapping of charge distributions across biological membranes and cell surfaces to investigate electrostatic interactions	Imaging in physiological saline solutions presents significant challenges	Requires conductive probe/non-conductive specimens/dry conditions or specific liquid media	1−10 nm (lateral resolution)/< 1 mV (potential resolution)	Minute-scale per image acquisition	Birkenhauer and Neethirajan 2014
SNOM	Nanoscale spatial resolution, label-free biological sample identification, single-molecule sensitivity	Weak signal intensity, stringent sample/probe requirements	Demanding sample flatness, requires functionalized probes	10−30 nm/< 100 nm	Minute-scale per image acquisition	Hu *et al*. 2023
TERS	Provides chemical specificity, enabling identification of individual protein secondary structures, DNA bases, and other targets	Probe preparation is challenging and suffers from poor reproducibility, probe/sample may be impaired by laser-induced heating Long imaging times	Requires Raman-active samples, requires metal-coated probes	Nanoscale spatial resolution	Minutes to hours per spectrum	Gao *et al*. 2018
SECM	Directly probes the functional dynamics in live cells, including metabolic activity, enzymatic catalysis, and transmembrane transport	Spatial resolution is confined by the probe dimensions	Requires electrolytic solution environment, compatible with non-conductive specimens	Determined by probe tip dimensions (resolution < 20 nm achievable)	Minute-scale per image acquisition	Conzuelo *et al*. 2018; Filice and Ding 2019

### AI-enhanced scanning probe microscopy techniques

While SPM excels at atomic-scale imaging and manipulation, its broader application is hindered by fundamental constraints. Inefficient data acquisition, operator-dependent reproducibility, probe instability, and complex analysis workflows all reduce the efficiency of experiments. The core challenge of SPM is that it relies heavily on people, requiring operators to use their judgment and intervene manually.

AI offers a path to overcome these limitations by transforming SPM from a manually operated tool into an autonomous scientific instrument. The ultimate goal is to automate the entire workflow — from parameter optimization and probe control to data acquisition and real-time interpretation. Such automation offers huge benefits that greatly improve the efficiency, consistency, and speed up scientific breakthroughs.

AI is already beginning to replace experience-dependent manual processes with quantifiable, automated workflows. This shift is exemplified by research where algorithms like Bayesian optimization autonomously identify optimal imaging parameters. By defining a clear image quality metric as a "reward function", these systems quickly explore a wide range of parameters, guaranteeing consistent, high-quality imaging and greatly improving experimental reproducibility across diverse conditions (Liu *et al*. 2025; Vasudevan *et al*. 2021).

Stable probe performance is fundamental to SPM operation. When real-time convolutional neural network (CNN) monitoring detects probe degradation — such as tip-induced image artifacts — an alert activates a deep reinforcement learning (DRL) protocol. This system autonomously generates and executes condition-specific corrective actions (*e*.*g*., voltage pulses), restoring optimal probe function without human intervention (Rashidi and Wolkow 2018).

DRL further enables AI to master precise atomic/molecular manipulation. By defining discrete states (molecular positions), actions (voltage pulses applied by the tip), and scalar rewards (proximity to target locations), AI autonomously acquires optimal manipulation policies through iterative exploration without explicit programming. Empirical studies demonstrate that AI-driven systems can successfully reposition individual molecules to desired sites at greater than 80% success rates, with capabilities extending to selective chemical bond cleavage. This breakthrough establishes a foundational platform for atomically precise fabrication and programmable surface synthesis (Ramsauer *et al*. 2023; Wu *et al*. 2025).

The integration of AI with SPM is bringing about a revolutionary change in scientific research. By liberating researchers from repetitive operational tasks, this approach enables researchers to refocus on higher-level conceptual challenges. Critical hurdles remain: overcoming data scarcity, enhancing model generalizability, and developing "multifunctional scientific agents" capable of comprehending abstract research objectives. Ultimately, this AI-driven SPM technology will fundamentally streamline traditional scanning processes. The technology dramatically improves scanning efficiency and eliminates dependence on operator expertise for atomic-scale manipulation. Crucially, AI-enabled adaptive systems make atomic-level characterization accessible across disciplines. This breakthrough will accelerate discovery and create new research opportunities.

## APPLICATIONS OF SPM TECHNOLOGIES IN DIFFERENT BIOLOGICAL FIELDS

### Nucleic acids (genetic information analysis)

With the widespread adoption of STM in the biological field, it has been successfully applied to observe biomacromolecules and has demonstrated feasibility for imaging in diverse environments, including solution, vacuum, and atmosphere. Albrecht (Albrecht 1989) and Driscoll *et al*. (Driscoll *et al.* 1990) successfully resolved double-stranded deoxyribonucleic acid (DNA) structures using atmospheric and UHV STM. Their work revealed the helical conformation and periodicity of DNA, correlating morphology with potential barrier height during imaging to interpret structural features of the DNA backbone, phosphate groups, and base pairs. These findings established STM as a promising tool for DNA characterization and sequencing applications.

Further advancements were made by Tanaka and Kawai *et al*., who deposited fluorescein isothiocyanate (FITC)-labeled DNA molecules onto a Cu(111) surface via a pulse injection method and performed phase-locked STS mapping (Tanaka and Kawai 2009; Yoshida *et al*. 2007). Comparative spectral analysis revealed distinct electronic signatures for purine and pyrimidine bases, marking a pivotal advancement toward practical DNA sequencing and providing mechanistic insights into the electronic basis of genetic information storage.

**Figure 3:**
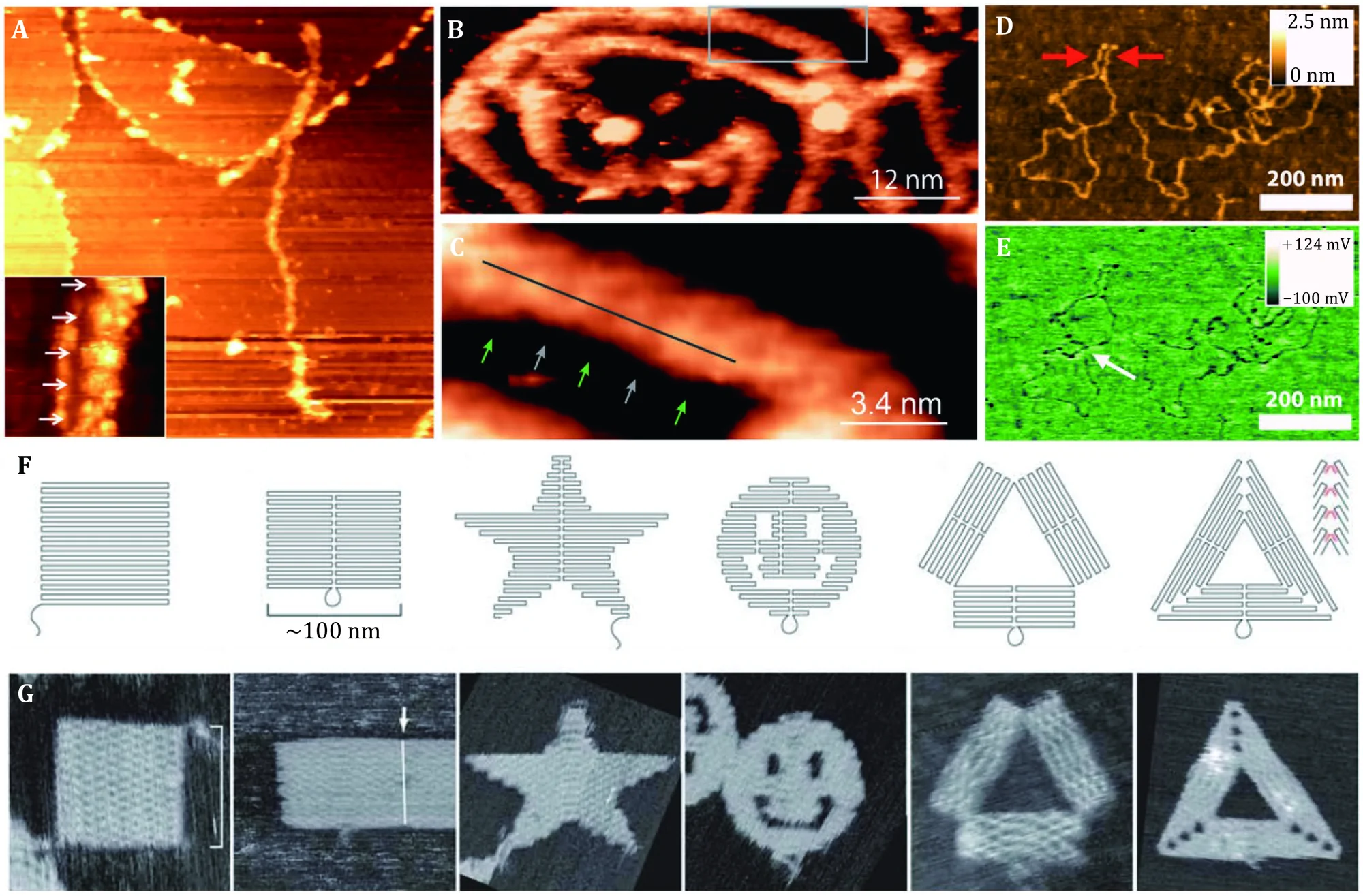
SPM observation images for the nucleic acids field. **A** STM image (200 nm × 200 nm) of a linear DNA deposited on Au(111) using an electrospray method. Inset: high-resolution STM image (20 nm × 20 nm) of the linear DNA (Terasaki and Yokoyama 2019). Structural modification of DNA studied by STM. **B**,**C** High-resolution imaging of dsRNA using AM-AFM (**B**), and Detail at higher magnification of Panel B (**C**) (Ares *et al*. 2016). **D**,**E** Topography (**D**) and KPFM (**E**) of single DNA molecules deposited on mica (Leung *et al*. 2010). **F**,**G** Folding paths of DNA origami shapes (**F**), and AFM images corresponding to DNA origami, all with dimensions of 165 nm × 165 nm (**G**) (Rothemund 2006)

STM also provides atomic-scale access to DNA topology in Fig. 3A. Studies observe DNA adopting surface-adsorbed conformations, such as straight, curved, or coiled, with conformational distributions exhibiting dependency on DNA concentration and surface pretreatment (Tanaka *et al*. 1999). STM can clearly reveal the detailed structure of the DNA double helix.

AFM high-resolution imaging technology was initially applied to nucleic acid molecules by Weisenhorn *et al*. (Weisenhorn *et al*. 1990), who successfully imaged a 20-base single-stranded DNA molecule. However, the initial sample preparation methods remained in an undeveloped state, resulting in poor DNA-substrate adhesion, which limited imaging quality. Vesenka *et al*. (Vesenka *et al*. 1992) and Bezanilla *et al*. (Bezanilla *et al*. 1993) pioneered modified substrate functionalization protocols, enabling robust DNA imaging under near-physiological conditions. Around the same time, Bustamante *et al*. (Bustamante *et al*. 1992) and Lyubchenko *et al*. (Lyubchenko and Shlyakhtenko 2009) validated reproducible ambient and liquid-phase AFM imaging of DNA, while Maeda *et al*. (Maeda *et al*. 1999) directly resolved the double-helical topology of DNA. With the maturation of AFM technology (Lyubchenko *et al*. 2011), high-fidelity imaging of structural polymorphisms in free DNA (Leung *et al*. 2012; Pyne *et al*. 2014), DNA-protein complexes (Gilmore *et al*. 2009; Lyubchenko and Shlyakhtenko 2009; Umemura *et al*. 2001), and RNA (Figs. 3B and 3C) became possible. These advancements provided critical insights into the structural stability and assembly mechanisms of nucleic acids (Ares *et al*. 2016; Mattick and Rinn 2015). Progressive refinements in instrumentation further enhanced spatial resolution (Figs. 3F and 3G), firmly establishing AFM as an indispensable nanoscale characterization platform for transformative biological nanotechnology, particularly in the field of DNA origami (Douglas *et al*. 2007; Qian *et al*. 2006; Rothemund 2006).

Natural RNA primarily exists as single-stranded polynucleotides that fold into complex tertiary structures based on their nucleotide sequences. Although chemically less stable than DNA, these folds give rise to a diverse repertoire of rigid structural motifs. Uroda *et al*. (Uroda *et al*. 2020) developed an integrated methodology that combines long non-coding RNA (lncRNA) purification with solution hydrodynamic analysis and single-particle AFM imaging. This pipeline produces highly homogeneous lncRNA preparations and resolves their 3D topological architectures at a resolution of approximately 15 Å. This represents a transformative advancement in RNA structural studies using AFM, enabling deeper insights into RNA folding and function.

Beyond STM and AFM, additional scanning probe methodologies have been developed to enable functional characterization of nucleic acids. For example, dual-frequency KPFM, applied to DNA adsorbed on insulating mica substrates, successfully resolves native-state surface potential profiles (Figs. 3D and 3E). This advancement establishes a critical foundation for investigating electrostatic phenomena in complex biological systems, such as DNA-protein complexes (Leung *et al*. 2010). By mapping surface potential at high resolution, dual-frequency KPFM provides valuable insights into the role of charge distribution in the structural and functional dynamics of nucleic acids.

TERS offers a powerful tool for the comprehensive structural analysis of DNA. Treffer *et* *al*. (Treffer *et al*. 2011) applied this technique to identify base-specific spectral markers within single-stranded calf thymus DNA. Furthermore, TERS explains adenine-silver interactions through a combination of density functional theory (DFT) vibrational calculations and TERS band shift analysis (Watanabe *et al*. 2004). For sequencing applications, TERS achieves single-base resolution by anchoring single-stranded DNA (ssDNA) from bacteriophage via phosphate groups to expose nucleobases. Spectral validation against reference sequences confirms the distinct spectral signatures of each base, enabling high-resolution imaging and sequencing of diverse genetic materials, including RNA (He *et al*. 2019, 2021).

### Proteins/peptides (biomacromolecule research)

**Figure 4:**
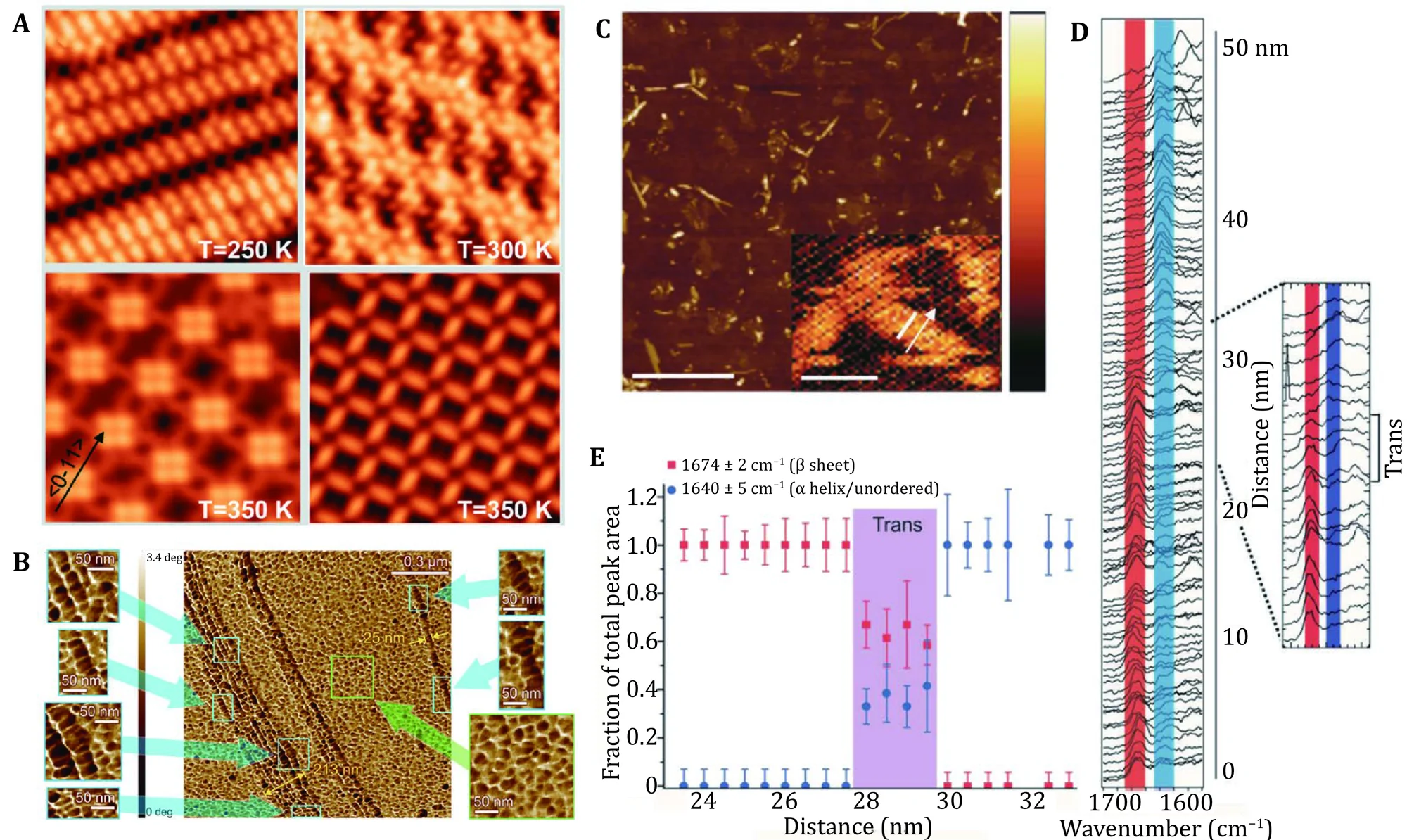
Nanoscale characterization of protein and peptide self-assembly using scanning probe microscopy. **A** STM image of different self-assembled structures produced by evaporating Glu on Ag(100) at different temperatures (image size: 80 Å × 80 Å) (Smerieri *et al*. 2011). **B** Noncontact mode AFM topography images showing silk protein structures prepared by spin-coating a 1:10 silk solution (Greving *et al*. 2012). **C** AFM image of human islet amyloid polypeptide (hIAPP) fibrils formed at pH = 2.0. Scale bar is 1 μm and height bar is 20 nm. Inset: AFM image obtained by scanning with a TERS AFM tip. The white line represents positions of the 100 TERS measurements that were recorded with 0.5 nm intervals along a line perpendicular to the fibril axis. Scale bar is 100 nm; the height bar represents 13.6 nm for the inset **D** The amide I region of the spectra. Red color indicates band position for β-sheets, and blue indicates unordered or α-helical structure. Right: enlarged transition region. Spectra showing mixed structures are indicated (“trans”). **E** Relative secondary structure content in terms of β-sheets (red squares) and unordered or α-helical structure (blue circles) for spectra in the transition region. The distance on the *x*-axis corresponds to the distance from the first measurement point in Panel C. Panels C−E were adapted from van den Akker *et al*. (2015)

STM is also a powerful tool for studying the self-assembly of protein subunits (amino acids and peptides) and the folding morphology of proteins. This technique enables the direct observation of various microscopic structures of amino acids and peptides on different surfaces, including molecular chains, island structures, layered structures, and two-dimensional ordered structures. Figure 4A provides a direct visual representation of the structural self-assembly process of amino acids and polypeptides (Smerieri *et al*. 2011; Xu *et al*. 2011). STM further enables molecular-scale characterization of lipid membrane packing geometries, precisely determining stacking architectures in pure fatty acid systems (Smith *et al*. 1987).

STS imaging reveals surface interaction-induced electronic state modifications in biomolecules (Ha *et al*. 2020), while electronic transport measurements validate structure−function relationships in peptide systems (Nguyen *et al*. 2020). These approaches are essential for elucidating molecular electronic properties and the charge transfer mechanism.

AFM imaging serves as a powerful tool for characterizing the structural composition, conformational dynamics, and self-assembly behaviors of bioactive proteins, offering valuable insights into their biological functions. Key applications include the study of morphological changes during Beta-lactoglobulin aggregation (Adamcik *et al*. 2010), the formation of monomer aggregates and fibril structures in fibronectin (Bergkvist *et al*. 2003; Chen *et al*. 2007; Lin *et al*. 2000), imaging of prion proteins under varying pH conditions (Wang *et al*. 2015), and high-resolution visualization of light-driven proton pump bacteriorhodopsin (BR) (Medalsy *et al*. 2011; Müller *et al.* 1999; Rico *et al*. 2011). Additionally, AFM has been employed to study alpha-synuclein (Sweers *et al*. 2012) and natural silk proteins (Fig. 4B) (Greving *et al*. 2012).

A key application of AFM is single-molecule force spectroscopy, which is used to investigate the mechanical properties and folding/unfolding kinetics of biomolecules. Since stable tertiary structures are essential for physiological function, controlled unfolding experiments in solution provide insights into conformational transitions and intramolecular forces. Rief *et al*.'s pioneering study in 1997 on the mechanical stretching behavior of titin established the foundation for single-molecule biomechanics (Rief *et al*. 1997). Later, Rico *et al*.'s development of high-speed force spectroscopy (HS-FS) enabled direct comparisons between molecular dynamics simulations and experimental results (Rico *et al*. 2013). AFM has also been extensively employed to examine the folding mechanisms of membrane proteins, such as bacteriorhodopsin (Kessler *et al*. 2006; Oesterhelt *et al*. 2000).

Beyond STM and AFM, KPFM has also been applied in the field of protein research. It can be used to investigate conformation-dependent surface potential shifts in *β*-lactoglobulin fibrils under varying pH conditions (Lee *et al*. 2012). Furthermore, KPFM enables quantification of kinase-ligand binding thermodynamics, including ATP interactions and antagonist-induced inhibition, providing valuable insights for rational drug design (Park *et al*. 2011). TERS achieves residue-specific mapping of amino acids in protein primary structures (Figs. 4C−4E) (Kurouski *et al*. 2014; van den Akker *et al*. 2015) while resolving conformational signatures of secondary structures (Lipiec *et al*. 2018, 2021; Szczerbiński *et al*. 2020). SECM further characterizes membrane permeability dynamics in live cells (Li *et al*. 2016; Roberts *et al*. 2013).

### Cells (cell biology research)

**Figure 5:**
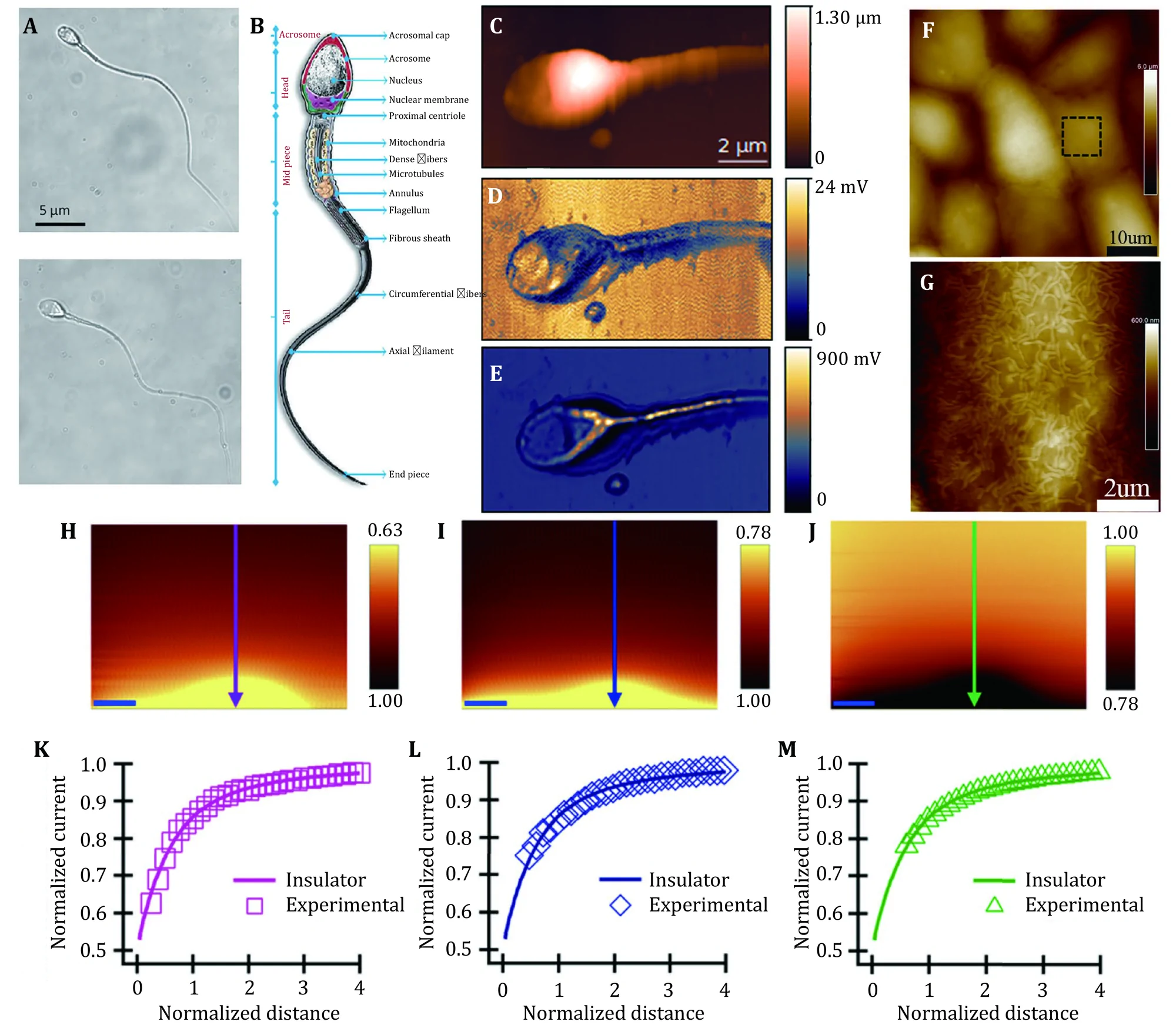
Nanoscale imaging of cellular structures and functions using scanning probe microscopy. **A** Human spermatozoa with normal features. Bright field optical image of spermatozoa with normal features as observed with a ×100 objective (Zeiss). **B** Sketch illustrating all the main sperm cellular structures as SNOM topography. **C** SNOM topography image. **D** SNOM reflection image. **E** SNOM transmission image. **F** PeakForce Tapping image of glutaraldehyde-fixed MDCK C11 cells (50 μm × 50 μm). **G** A higher resolution image (8 μm × 8 μm) in the area indicated by the black outline in Panel F. **H**−**M** Typical SECM depth scan images of single T24 collected using redox mediators: FcCOO^−^ (**H**), Fc(COO)_2_^2^^−^ (**I**), and Ru(NH_3_)_6_^3^^+^ (**J**). The scale bar in each SECM image represents 10 μm. The corresponding experimental PACs extracted at each arrow in Panels H−M are overlaid onto theoretical PACs generated in an insulating cell, where the redox mediator is FcCOO^−^ (purple) (**K**), Fc(COO)_2_^2^^−^ (blue) (**L**), and Ru(NH_3_)_6_^3^^+^ (green) (**M**). Please note, the color map (current) for Ru(NH_3_)_6_^3^^+^ in Panel J appears inverted from the ferrocene carboxylates in Panels H and I, due to the opposing redox chemistry occurring at the UME tip Panels A−E were adapted from Troian *et al*. (2020), Panels F and G were adapted from Schillers *et al*. (2016), and Panels H−M were adapted from Li *et al*. (2016)

Researchers have utilized AFM technology to observe live platelets (Radmacher *et al*. 1992) and filamentous actin (F-actin) in living cells (Henderson *et al*. 1992). It has been demonstrated that AFM can directly resolve the ultrastructure of live cells under near-physiological conditions. Since then, AFM technology has been widely applied to the fine structural characterization of biological cells, such as studies on the actin cortex of live cells (Eghiaian *et al*. 2015), imaging of single tumor cells (Li *et al*. 2015a), and imaging of microvilli structures in epithelial cells (Figs. 5F and 5G) (Schillers *et al*. 2016). Quantitative analysis of cell membrane structural parameters, such as cell size, adhesion force, and surface roughness, provides a reliable means to differentiate diseased cells from normal ones (Lee *et al*. 2015; Zhang *et al*. 2012). Additionally, this approach enables pharmacological evaluations by monitoring drug-induced biomechanical changes in cancer cells (Kim *et al*. 2012; Pi *et al*. 2015), including alterations in cell membrane morphology (Wang *et al*. 2009), adhesion force, and surface roughness (Shi *et al*. 2015), which are valuable for assessing the anti-cancer activity of drugs. For example, AFM imaging of esophageal epithelial cells can determine functional integrity based on surface roughness measurements. Beyond eukaryotic cells, AFM is also widely applied in prokaryotic cell research — investigating the size, structure, and assembly of bacterial surface proteins in single live bacteria (Fantner *et al*. 2010; Turner *et al*. 2018).

Real-time analysis of AFM single-molecule force spectroscopy curves provides valuable nanoscale mechanical information, including deformation maps, adhesion force maps, and Young's modulus. The mechanical properties of live cells are closely linked to their physiological functions. This technique has been employed to measure the mechanical properties of various live cells, such as red blood cells (Picas *et al*. 2013), kidney cells (Matzke *et al*. 2001), brain endothelial cells (Végh *et al*. 2011), and bacterial pathogen cells (Carrasco *et al*. 2011; Roos *et al*. 2009), enabling the study of cellular physiological and pathological functions. In recent years, single-molecule force spectroscopy has been used to quantify the expression of specific biomolecules on the cell surface (Knoops *et al*. 2018) and has been integrated with molecular biology techniques to investigate adhesion properties and elasticity (Beaussart *et al*. 2013; Feuillie *et* *al*. 2018; Mathelié-Guinlet *et* *al*. 2020; Sullan *et* *al*. 2015).

In addition to the above two techniques, numerous functionalized scanning probe techniques have also been widely applied in the cellular field. SNOM has already been successfully applied in reproductive medicine for ultrastructural analysis of sperm (Figs. 5A−5E) (Troian *et al*. 2020), as well as for the structural analysis of biological membranes (Dickenson *et al*. 2010). *In situ* monitoring also has a wide range of applications with SECM. For example, the effect of divalent cadmium on the membrane permeability of bladder cancer (T24) cells was monitored using various redox mediators (Figs. 5H−5M) (Li *et al*. 2016). The non-destructive and non-invasive advantages of SECM in single-cell studies make it an important tool for investigating the effects of metal ions on cell membrane permeability (Filice *et al*. 2019). Additionally, some research teams have used antimony microelectrodes as SECM probes to image the pH distribution of gelled yeast cells (Horrocks *et al*. 1993).

### Widespread applications of SPM in other biological fields

SPM technology is now being widely used in many areas of biological research. It can not only investigate genetic information, biomolecules, and cell biology, but also contribute to diverse fields, including disease diagnosis, drug screening, neural signal analysis, biosensing, and biological imaging. In the field of biomolecular science, SPM has been invaluable in studies on carbohydrates (Morris *et al*. 2011; Vikas *et al*. 2021), viruses (Roos *et al*. 2010), and lipid bilayers (Redondo-Morata *et al*. 2012). In the medical field, SPM also plays a key role in the diagnosis of diseases like cancer and anemia (Kwon *et al*. 2019; Lee *et al*. 2015; Zhang *et al*. 2012), as well as in evaluating drug effects (Pi *et al*. 2015). In the field of plant biology, SPM can be used to study the nanostructure of cell walls (Pu *et al*. 2025) and dynamic processes like growth, maturation, modification, and aging (Li and Kasal 2025). SPM can also reveal the mechanical heterogeneity of different regions of the cell wall. It can map how a cell's stiffness changes dynamically as it grows and precisely quantify how genetic mutations or chemical treatments impact its nanomechanical properties. This provides "mechanical coding" insights that go beyond traditional macroscopic tests (Charrier *et al*. 2018), significantly deepening our understanding of the relationship between plant cell wall structure and function. Beyond plant tissues, AFM-based force spectroscopy extends to viruses, quantifying capsid mechanics and probing receptor–ligand interactions to connect nanoscale forces with entry behavior.

## SUMMARY AND OUTLOOK

SPM has diversified rapidly, with innovations spanning both imaging and spectroscopy. Imaging modalities include STM, which resolves atomic-scale surface structure via quantum tunneling, and AFM, which provides high-resolution topography — and, in liquid, gentle imaging of soft biological samples. They are complemented by KPFM mapping surface-potential distributions and SNOM achieving sub-diffraction optical images that capture nanoscale signatures of cellular structures. Spectroscopic and functional probes extend this toolkit: TERS provides chemically specific, nanometer-resolved Raman spectra, SECM visualizes local redox activity, and AFM-based force spectroscopy quantifies receptor–ligand binding forces, protein/DNA unfolding pathways, and cell adhesion and elasticity, linking mechanical response to biochemical state. In parallel, EM (including cryo-EM) remains central to biological structure determination; SPM complements EM by offering label-free, surface-specific, and in-liquid functional and mechanical readouts. Correlating TEM’s near-atomic structural maps with SPM’s local physicochemical and biomechanical measurements yields a more complete picture — even in EM-centric workflows, the two together propel biological research forward. Looking ahead, integrating AI enables fully autonomous, closed-loop workflows in which an agent designs and executes experiments, analyzes data in real time, and iteratively optimizes protocols — broadening the scope and depth of biological inquiry.

Despite these advancements, characterizing biological samples with SPM remains a significant challenge, and its full potential is yet to be realized. On the technical front, one of the most formidable yet promising challenges is achieving true *in vivo* imaging. As a surface-probing technique, SPM relies on maintaining an extremely close proximity between probe and sample for imaging, which fundamentally differs from optical methods capable of penetrating tissue. This limitation presents a major obstacle to deep *in vivo* imaging. As a result, most current "live" studies are restricted to *in vitro* cell cultures or *ex vivo* tissue slices. While these studies provide valuable insights, they cannot fully replicate cellular behavior within the native physiological microenvironment. Moreover, the application of extreme conditions — such as ultra-low temperatures or strong magnetic fields — to biological studies is still relatively new, although these techniques are already well-established in materials science. In the future, SPM technology is evolving toward greater multi-functionality, intelligence, and automation. This progress is expected to enable groundbreaking applications, including true *in vivo* imaging, multi-probe cellular microsurgery, and even precise *in situ* gene editing. Ethical considerations are crucial for advancing the use of SPM technology in the biomedical field. Researchers must ensure transparency and strict compliance with ethical review protocols, including obtaining participants' documented informed consent that clearly discloses associated risks and uncertainties. By prioritizing these core elements, we can ensure this potentially transformative technology benefits humanity in a responsible way.
